# The Textile Plot: A New Linkage Disequilibrium Display of Multiple-Single Nucleotide Polymorphism Genotype Data

**DOI:** 10.1371/journal.pone.0010207

**Published:** 2010-04-27

**Authors:** Natsuhiko Kumasaka, Yusuke Nakamura, Naoyuki Kamatani

**Affiliations:** 1 Center for Genomic Medicine, RIKEN, Tokyo, Japan; 2 The Institute of Medical Science, The University of Tokyo, Tokyo, Japan; Erasmus University Medical Center, Netherlands

## Abstract

Linkage disequilibrium (LD) is a major concern in many genetic studies because of the markedly increased density of SNP (Single Nucleotide Polymorphism) genotype markers. This dramatic increase in the number of SNPs may cause problems in statistical analyses, such as by introducing multiple comparisons in hypothesis testing and colinearity in logistic regression models, because of the presence of complex LD structures. Inferences must be made about the underlying genetic variation through the LD structure before applying statistical models to the data. Therefore, we introduced the textile plot to provide a visualization of LD to improve the analysis of the genetic variation present in multiple-SNP genotype data. The plot can accentuate LD by displaying specific geometrical shapes, and allowing for the underlying haplotype structure to be inferred without any haplotype-phasing algorithms. Application of this technique to simulated and real data sets illustrated the potential usefulness of the textile plot as an aid to the interpretation of LD in multiple-SNP genotype data. The initial results of LD mapping and haplotype analyses of disease genes are encouraging, indicating that the textile plot may be useful in disease association studies.

## Introduction

Advances in high-throughput genotyping technology have enabled us to identify remarkably dense SNP (Single Nucleotide Polymorphism) genotype markers on human chromosomes [1]. Linkage disequilibrium (LD) is a topic of interest because it impacts the search for disease-susceptibility loci in genome-wide association studies [2], [3] and can reveal underlying historical and biological processes, such as selection [4], [5], mutation [6], recombination [7], [8] and population history [9].

Graphical representations of LD for multiple-SNP genotypes have been developed to assess the presence of LD in practical data sets. Various pairwise LD statistics, such as 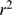 or 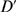 (reviewed in, *e.g.*, [10]), can be shown by triangular heat map displays [11] in which the color shading indicates the strength and distribution of the pairwise LD. A segment with consistently high LD, a so-called *LD block*, is visually apparent in such displays. From these displays, it is clear that LD is discontinuous and heterogeneous over entire human chromosomes [7]. To incorporate such heterogeneity into further genetic and statistical analyses, the visualization of pairwise LD is now being recognized as a way to maximize insight into the LD present in multiple-SNP genotype data.

Isometricblocks and bifurcation plots [12] are alternative approaches to visualizing and understanding LD among multiple SNPs via population haplotypes that provide powerful displays of LD and its breakdown with increasing distances between markers. Such displays show the direct association of alleles among multiple-SNP loci by means of connecting lines made up of population haplotypes and their frequencies inferred from the sampled SNP genotypes with statistical algorithms, such as EM (Expectation-Maximization algorithm, [13]).

The textile plot [14] is another way to visualize LD among multiple-SNP genotype data. It is essentially a parallel coordinate display [15], [16], but both quantitative and qualitative data (including SNP genotypes) or a mixture of these different data types can be accommodated within the plot. In addition, the locations and scales of whole axes are optimally chosen so that the connecting lines, each of which represents an observation, are aligned as horizontally as possible. Hence, it can graphically represent the linearity and orthogonality of high-dimensional (multi-variate) data. The algorithm employed in the textile plot is closely related to principal component analysis (PCA) or multiple correspondence analysis (MCA: also known as homogeneity analysis [17]); all of these methods aim to assign the optimal geometrical configuration to variables and data points in a low-dimensional linear space. The major difference between this method and PCA or MCA is that the textile plot employs a parallel coordinate system in which SNPs are aligned from left to right according to their physical order on a chromosome so that the LD can be directly shown by queues of SNP genotypes. Therefore, the appearance of the plot is more similar to isometric blocks or a bifurcation plot than to a heat map display.

An advantage of the textile plot is that it does not assume any statistical or probabilistic models, so inference of the population haplotypes or frequencies from the sampled genotype data is unnecessary. The resulting plot is rather heuristic compared with the other graphical representations mentioned above, which assume Hardy-Weinberg equilibrium and require haplotype inference. The textile plot simultaneously captures information about several genetic variations among multiple-SNP genotypes, such as LD and haplotype structures, in one display, and such results are usually compatible with those of confirmatory analyses based on probabilistic and statistical models. Our preliminary LD mapping results and haplotype analyses of disease genes have also been encouraging, thereby suggesting the usefulness of the plot in disease-association studies. Here, we report the results of our first attempt to introduce the textile plot into genetics and disease association studies.

## Results and Discussion

### LD between adjacent SNPs

**Figure 1:**
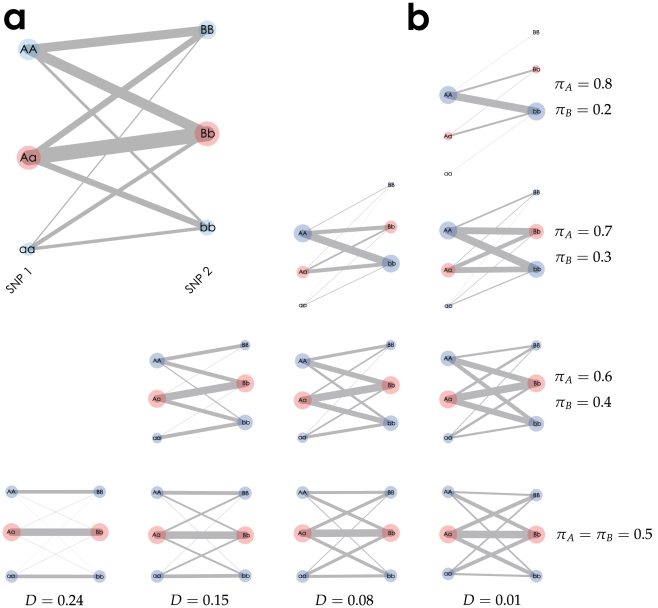
Textile plots of simulated two SNP genotype data. (a) The plot corresponds to the 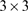 contingency table (Table 1) whose marginal genotype counts are shown by each circle with area which is proportional to the number of replicates, and each pairwise count of the two genotypes is shown by the width of a segment connecting two genotypes. (b) The column indicates the difference of the covariance 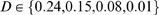 and the row indicates different pair of allele frequencies 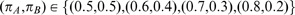. The greater the grade of the line crossings between two SNPs, the weaker the covariance between the SNPs. We also see that the steeper the slope between heterozygotes, the greater the allele frequency difference between *A* and *B*.

We began by visualizing two SNP loci on a chromosome, with alleles *A* and *a* at the first locus and with *B* and *b* at the second locus. Figure 1a shows a textile plot for two-SNP genotype data where the combinatorial and marginal genotype counts are shown in a 3
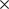
3 contingency table (Table 1) that was simulated under Hardy-Weinberg equilibrium (HWE) with fixed haplotype frequencies: 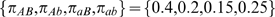. On the textile plot, the genotype at each locus is represented by a circle on a vertical axis with the area proportional to its count (*e.g.*, the smallest circle with a count of 1,465 corresponds to the genotype *aa* at SNP locus 1), and pairs of genotypes between the adjacent SNPs are connected by a segment with its width proportional to the number of replicates associated with the pair of genotypes (*e.g.* the thickest line with a count of 2,583 lies between genotypes *Aa* at SNP locus 1 and *Bb* at SNP locus 2).

**Table 1:** Simulated data for two SNP loci.

		SNP 2			
		BB	Bb	bb	
	AA	1,567	1,757	472	3,796
SNP 1	Aa	1,091	2,583	1,065	4,739
	aa	210	696	568	1,465
		2,859	5,036	2,105	10,000

Genotype counts for two SNP loci were simulated under HWE with fixed haplotype frequencies 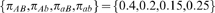.

The most important aspect of the textile plot is the introduction of an optimal criterion, the so-called *horizontalization criterion* (see Methods for more details), so that all of the connecting lines between the SNPs are aligned as horizontally as possible. The introduction of such a criterion allows for the observation of LD between adjacent SNPs by specific geometrical shapes. The textile plot shows the sign and intensity of the covariance 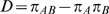 by the allele locations and the degree of line crossings between the two loci. Here, 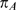 and 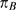 indicate the allele frequencies of *A* at the first locus and of *B* at the second locus, and 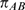 is the frequency of the haplotype with the *A* and *B* alleles. As shown in Figure 1a, the covariance is positive (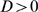) because of the relative positions of the genotypes between the two loci (*i.e.*, genotype *AA* is vertically closer to *BB* than to *bb*). In addition, 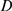 is small (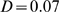) because many line intersections exist between the genotypes of the two SNP loci. The plot also shows the allele frequency differences (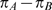) for 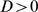 (or 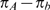 in the case of 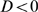) by the angle of the connecting line between the heterozygotes. As shown in Figure 1a, 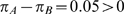 because the plot angles upward from *Aa* to *Bb*. Figure 1b summarizes the relationship between the textile plots and various LD and allele frequency settings. The difference in the allele frequencies between the two SNPs decreases from the top to bottom, and the strength of the LD decreases from the left to right (Table S1 provides the corresponding 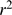 and 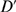 statistics).

**Figure 2:**
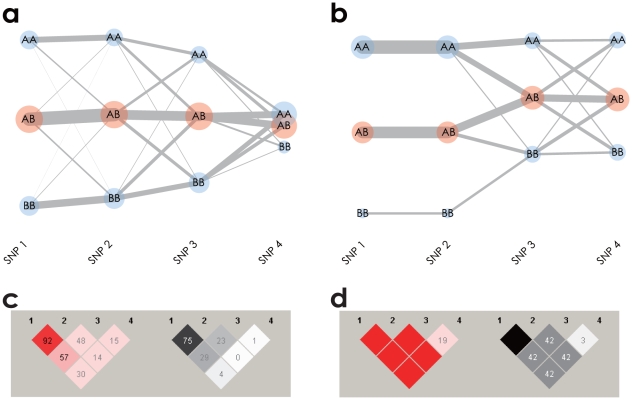
Textile plots and pariwise LD displays for simulated data. (a) Textile plot shows LD between adjacent SNPs by means of crossing lines which tend to increase if the adjacent SNPs are in linkage equilibrium (LE) and decrease in LD. (b) Textile plot shows the absolute and complete LD by specific geometrical shapes. There exist the absolute LD between 1st and 2nd SNPs and the complete LD between 2nd and 3rd SNPs in contrast to the LE between 3rd and 4th SNPs. (c) Pairwise 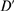 (left) and 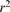 (right) displays of the same data shown in (a). (d) Pairwise 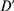 (left) and 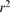 (right) displays of the same data shown in (b).

The interpretation of the LD between the two SNPs in the textile plot was generalized for multiple-SNPs. Figure 2a shows a textile plot of four SNPs simulated under HWE. A decreased LD strength between adjacent SNPs from left to right is clearly shown by increased line intersections between the SNPs. The textile plot also accentuates extreme LD structures by specific geometrical shapes. Figure 2b shows a textile plot of another simulated SNP data set in which the horizontal alignment of all three connecting segments between SNP1 and SNP2 represents the absolute LD (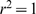 and 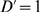). The connecting segments with a line intersecting only between a homozygote and the opposite side of a homozygote (*i.e.*, *AA* to *BB* between SNP2 and SNP3) represent the complete LD (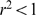 and 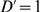), while the many line intersections between SNP3 and SNP4 indicate the linkage equilibrium (LE). Although those LD features are shown in pairwise LD displays in Figure 2c and 2d, the textile plot describes different aspects of LD in a single display.

**Figure 3:**
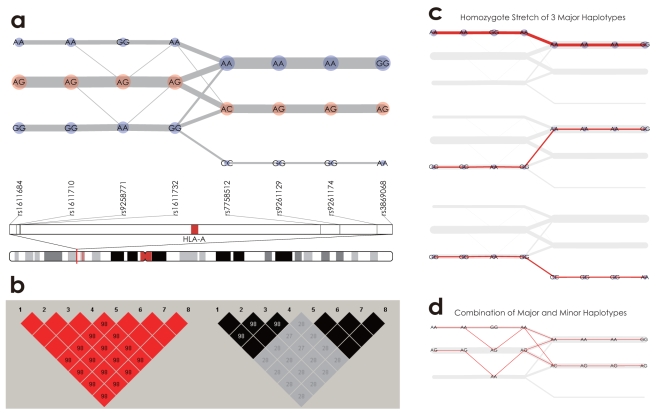
Textile plots and pairwise LD displays of 8 SNPs located in the region 29,933–30,112Kb on chromosome 6. (a) There exist two absolute LD blocks composed of the first 4 SNPs and the last 4 SNPs, and the 8 SNPs comprise a complete LD block as a whole. (b) The 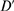 and 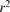 displays. (c) Homozygous pairs of major haplotypes *AAGAAAAG*, *GGAGAAAG* and *GGAGCGGA* are highlighted. (d) Heterozygous pairs of the major and a few minor haplotypes are highlighted.

To better understand the textile plot, we introduced SNP genotype data obtained from the BioBank Japan Project [18] for 934 Japanese individuals. Figure 3a shows a textile plot of eight SNPs located in a region (29,933–30,112Kb on chromosome 6). There are two absolute LD blocks, consisting of the first four SNPs and the last four SNPs; these eight SNPs comprise the complete LD block. However, the first LD block is not in absolute LD because of the connecting lines between the homozygotes and heterozygotes (*e.g. AA*-*AG* between the second and third SNPs from the left), indicating that the first four SNPs are in complete LD (because of the connecting line and the absence of a line intersection), consistent with the heat map displays shown in Figure 3b.

### LD among multiple SNPs

Because the textile plot employs a parallel coordinate system, the plot allows for the interpretation of LD between adjacent SNPs, but not between all pairs of SNPs (unlike the heat map display). However, because of the horizontalization criterion (see Methods for details), the textile plot can accentuate the underlying haplotype structure by displaying the diplotype configurations. This remarkable feature of the plot allows for the interpretation of LD among multiple SNPs in terms of haplotypes.

For example, according to the LD structure shown in Figure 3a, three major haplotypes (*AAGAAAAG*, *GGAGAAAG* and *GGAGCGGA*) can be inferred by paying attention to the diplotype configurations composed of a line of homozygotes (a homozygous stretch) among the eight SNPs (Figure 3c). Moreover, by subtracting the homozygous stretch from the textile plot, we could distinguish the existing minor haplotypes from the remaining diploid pairs of the major and minor haplotypes. For example, Figure 3d shows that the diplotype *AG*-*AG*-*AA*-*AG*-*AC*-*AG*-*AG*-*AG* is composed of the major haplotype *GGAGCGGA* and the minor haplotype *AAAAAAAG*. The textile plot is not a haplotype phasing algorithm and thus is not comparable to other specific algorithms [13],[19],[20]. Therefore, we became interested in how best to estimate the underlying haplotype frequencies with the textile plot. The square roots of the homozygous counts divided by the total sum are similar to the haplotype frequencies estimated by the EM algorithm (Table S2). Assuming HWE with large samples, the square root of the homozygous stretch count should be proportional to the population haplotype frequency. The textile plot could give a good approximation of the underlying haplotypes and their frequencies without any haplotype inference algorithm, although the estimation will not be as reliable with increasing numbers of haplotypes and decreasing LD strength.

**Figure 4:**
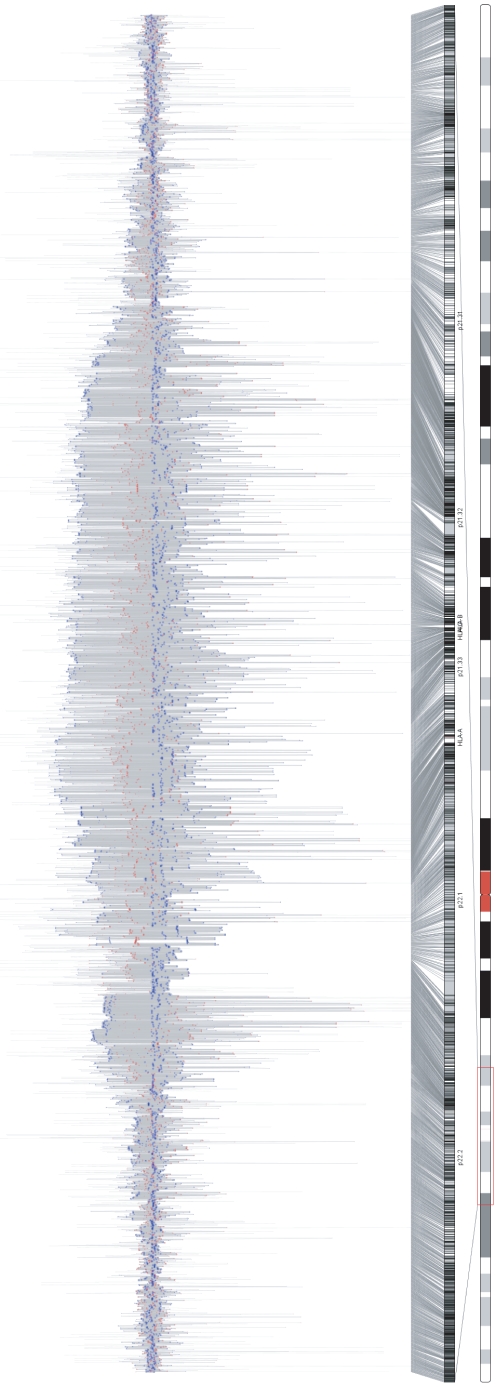
The textile plot of 3,736 SNPs (within 22,009,637–39,115,518 on chromosome 6) located in a region surrounding Major Histocompatibility Complex (MHC) region. It clearly shows the existence of very long range LD which covers the whole MHC region (the long range LD block can also be confirmed in the pairwise LD display based on the correlation coefficient 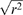 in Figure S2.

The horizontalization criterion was also included so that the vertical dispersion of the genotypes on each axis could provide information about the LD among SNPs spanning megabases with multiple blocks. The greater the dispersion of the genotypes between one homozygote and the other, such as 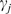 on the vertical axis 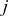 (Figure S1), the more likely the SNP is in LD with all of the other SNPs. On the other hand, a value of 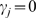 was considered as evidence that the SNP 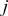 is in LE with the other SNPs. As shown in Figure 2a, the vertical dispersion of the SNPs decays from left to right with decreasing LD strength, and the rightmost SNP is barely visible above the axis because of its weak LD with the other SNPs. The lines of the expanded axes in Figure 4 also indicate a long-range LD block consisting of thousands of SNPs located in the MHC (Major Histocompatibility complex) region on chromosome 6, which was confirmed in the pairwise LD display based on the correlation coefficient 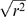 (Figure S2).

Note that, the size of 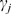 is globally determined according to the number of markers in LD with SNP 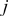 in the data set. Therefore, the appearance of the plot will dramatically change with the introduction of additional SNPs or a different SNP set even in the same LD block. Moreover, the plot does not incorporate any physical distance between SNPs, the dispersion on axis 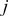 does not represent the physical size of the LD block nor the absolute scale of the strength of the LD block (see *e.g.*, Figures S3 and S4).

### Application to an association study

We assessed the utility of the textile plots for disease association studies using SNP genotypes recently reported in a genome-wide association study (GWAS) of chronic hepatitis B (BCH) in an Asian population [21]. The data consisted of 607 cases (in the second stage of the GWAS) and 934 controls (in the first stage of the GWAS). The SNPs were genotyped by the commercial platforms (Illumina and Affymetrix). In addition, the SNPs in exon 2 of the *HLA-DPA1* and *HLA-DPB1* genes were also genotyped by direct sequencing.

Unlike the Manhattan plot in the GWAS, the textile plot is less suitable for detecting disease susceptibility genes or SNPs from the vast number of genome-wide genetic markers. We began by looking at the LD in the entire MHC region (22–40Mb on chromosome 6) which had been already reported to be tightly associated with BCH [21]. The textile plot revealed the existence of a large LD block in the region that extends over 28–34Mb on chromosome 6 (Figure S5). The block is composed of several sub-blocks in tight LD, and the largest block is located in the region surrounding the *HLA-DPA1* and *HLA-DPB1* genes, consistent with the heat map displays (Figure S6). Therefore we focused on the SNPs surrounding the *HLA-DPA1* and *HLA-DPB1* genes. Direct sequencing of exon 2 in the *HLA-DP* genes revealed that, more than half of the SNPs were rare variants; in addition, a tri-allelic locus (DPB1_180) also exists (Figure S7). To focus on the interpretation of LD on the textile plot, here we further narrowed down the SNPs in this region to study the relationship between BCH and the very common SNPs for simplicity (*i.e.*, minor allele frequency 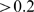).

**Figure 5:**
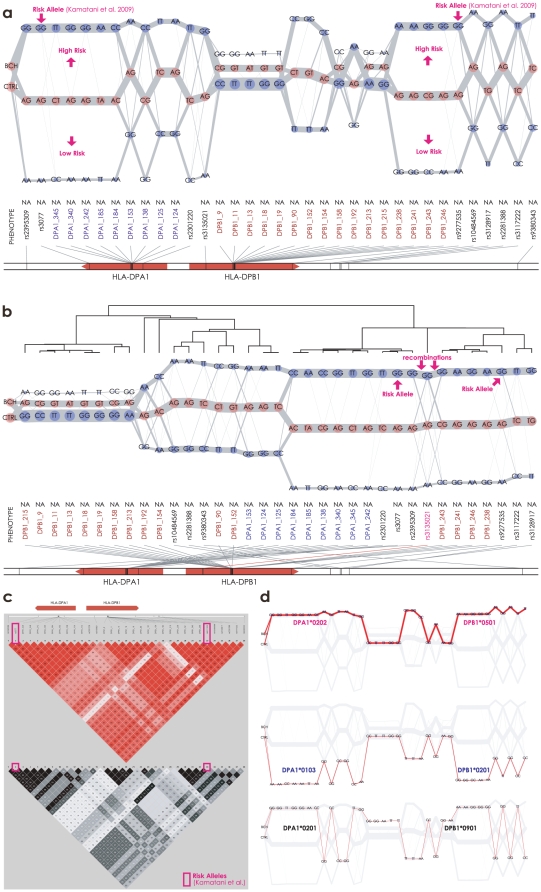
Textile plots and pairwise LD displays of SNPs located in a region surrounding *HLA-DP* genes, including the disease-susceptibility SNPs rs3077 and rs9277535 for the chronic hepatitis B. (a) The order of SNPs was according to the physical order on chromosome 6. The leftmost axis indicates the affection status for the chronic hepatitis B cases (BCH), and the controls (CTRL). (b) The order was rearranged by the heuristic clustering algorithm described in Methods. The affection status was not included in the clustering algorithm. (c) Pairwise 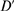 (top) and 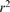 (bottom) displays. (d) The top 3 frequent homozygote stretches in *HLA-DP* region are highlighted.

The plot is rather useful for understanding the relationship between the multiple SNPs within a relatively small LD block and the phenotype(s). Figure 5a shows that pairs of adjacent SNPs are approximately in either absolute or complete LD because the typical geometrical shapes of extreme LD appeared. The leftmost axis indicates the affection status for the chronic hepatitis B cases (BCH) and the controls (CTRL). The locations of the affection status were optimally chosen by the same criterion used for selecting the vertical locations for each SNP (see Methods for more details). Therefore, a higher level of BCH than of CTRL indicates that the connecting lines of all of the BCH cases lie relatively higher than those of the CTRL subjects, suggesting that the genotypes located higher in the textile plot may induce the disease, while the genotypes located lower may not. In particular, the two SNPs with the highest associations in [21] are rs3077 (*A* 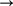 *G*) and rs9277535 (*A* 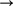 *G*), and the *GG* genotype of rs3077 (or rs9277535) was located above *AG*, and *AG* was located above *AA*, in accordance with the affection status reporting allele *G* as the risk allele for chronic hepatitis B.

Figure 5b provides another view of the underlying LD structure among the SNPs. Here, the set of SNPs incorporated into the textile plot is the same as in Figure 5a, but the order of the SNPs has been rearranged by the heuristic clustering algorithm (see Methods for more details). According to the textile plot and the supportive dendrogram on the plot, the set of SNPs is roughly summarized by three LD blocks across the genes. Each block is almost in absolute LD, and as a whole, they were in complete LD. Although the heat map displays in Figure 5c imply the existence of remote SNPs in tight LD across the two genes, the textile plot provides a much clearer view of the remote SNPs by a simple rearrangement of the SNP ordering. The disease susceptibility SNPs (rs3077 and rs9277535) are located a long distance away in Figure 5a but are in the same block in Figure 5b. Pairs of SNPs in this block are in almost absolute LD (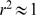), implying that all of the SNPs in this block have equivalent possibilities of disease susceptibility for BCH. However, there are several line intersections between rs2396309 and DPB1_243 and between DPB1_90 and DPA1_152 in Figure 5b, which might be due to recent recombination events occurring between the *HLA-DPA1* and *HLA-DPB1* genes. The two SNPs, rs3077 in *HLA-DPA1* and rs9277535 in *HLA-DPB1*, could be independently considered to be possible causative SNPs for this disease.

The plot also facilitates an understanding of the association between the *HLA-DP* alleles and susceptibility to BCH by means of the diplotype configurations. As shown in Figure 5d, the most frequent homozygous stretch is composed of DPA1*0202 and DPB1*0501; the second most frequent homozygous stretch consists of DPA1*0103 and DPA1*0201; and the third most frequent homozygous stretch running in between the previous two homozygous stretches made up of DPA1*0201 and DPB1*0901 (see Tables S3 and S4 for the mapping of the SNPs to the *HLA-DP* alleles). The vertical location of each *HLA-DP* allele clearly shows its effect on BCH in parallel with the affection status at the leftmost axis. These results are consistent with the haplotype analysis reported in [21] in which the haplotype of the first homozygous stretch is strongly associated with susceptibility to BCH, the second haplotype shows a protective effect against BCH and the third haplotype is a balance of the previous two haplotypes. The six HLA alleles forming the three haplotypes in Figure 5d were determined based on IMGT/HLA databases [22], so statistical inference was unnecessary. The detailed haplotype frequencies determined by the EM algorithm for the same dataset (HLA alleles along with the surrounding SNPs) were reported in [21].

### Concluding remarks

The observations reported herein are preliminary and were made after an initial exploratory analysis of the SNP genotype data using the textile plot. Further investigation based on statistical models is required to investigate these issues in more detail.

Nevertheless, the textile plot allows for a deeper understanding of LD and the underlying haplotype structure among SNP genotypes. Because of the introduction of the horizontalization criterion, the degree of horizontalness provides an interpretation of LD between adjacent SNPs by means of the line intersections. The two extreme cases of LD, absolute LD and complete LD, are represented by specific geometrical shapes. In addition, the LD among the overall SNPs can be captured by the vertical dispersion in the textile plot, which approximates the correlation structure for a number of SNPs. The direct presentation of the diplotype configurations in conjunction with such LD information allows for the simultaneous inference of the underlying haplotype structures in a single display.

The results of the initial LD mapping and haplotype analysis of disease genes associated with chronic hepatitis B were also encouraging, suggesting the usefulness of the plot in disease association studies. A heuristic algorithm to rearrange the SNP ordering was also introduced so that the textile plot can handle the LD between a pair of remote SNPs. The algorithm was able to summarize a complicated LD structure into a much simpler view in which the SNPs are clustered into several tight LD segments. Although the algorithm disrupts the physical position of the genetic markers and restricts biological interpretations, these results suggest another potential usage of the textile plot in association studies. Our method could be a useful way to visually select representative SNPs from each of the LD segments as non-redundant tag SNPs [23]. Moreover, the number of independent tests within each LD block may be systematically determined using the eigenvalues obtained by solving the optimization problem in the textile plot. In fact, SNPSpD (Single Nucleotide Polymorphism Spectral Decomposition, [24]) has already been proposed to estimate the number by using the eigenvalues of the correlation matrix of an LD block. It calculates the proportional reduction in the number of makers by the ratio of the eigenvalue variance to its maximum. It would be ideal if the textile plot could automatically reduce the volume of SNPs, both as an aid to the subsequent statistical analyses and to overcome the graphical limitation of the textile plot itself (*i.e.*, we may be able to handle only hundreds of SNPs in one display at a time).

**Figure 6:**
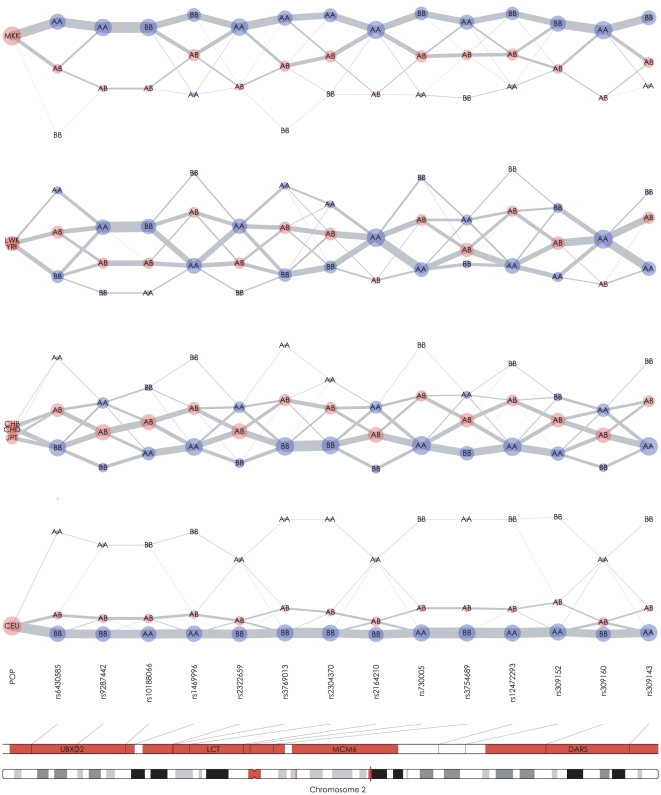
Textile plots of 14 SNPs surrounding *LCT* gene on chromosome 2 for the seven ethnic groups from HapMap data. Each of the four panels shows the plot of a specific population(s) indicated by the leftmost axis.

Although we focused on the interpretation of the LD and haplotype structure in the textile plot, but it may also be useful in displaying underlying historical processes, such as population mixing and admixing or positive selection. For example, Figure 6 shows a series of textile plots using fourteen SNPs surrounding the *LCT* gene on chromosome 2. The SNP genotypes for seven populations were obtained from the International HapMap Project [2]. The seven HapMap populations used in this analysis were classified into four groups according to the similarity of the haplotype structures in this region. The MKK (Maasai inKinyawa, Kenya) population contains haplotypes mainly from the upper alleles in the plot (*i.e.*, *AABBAA…* from the left), while the haplotypes of the CEU (Western European ancestry from the CEPH collection) population mainly consist of alleles from the opposite side (*i.e.*, *BBAABB…* from the left). The haplotype structures of five other populations YRI (Yoruba in Ibadan, Nigeria), LWK (Luhya in Webuya, Kenya), CHB (Han Chinese in Beijing, China), CHD (Chinese in Metropolitan Denver, Colorado) and JPT (Japanese in Tokyo, Japan) lie in between these extremes. However, the haplotype structures of YRI and LWK look similar to that of MKK, as expected, and the structures of CHB, CHD and especially JPT seem to be closer to that of CEU. Although heat map displays can reveal detailed information about the distributions of the pairwise LD for each of the populations (Figure S8), the textile plot accentuates the essential differences of the populations in this region. Therefore, it would be interesting to compare the textile plot with the principal component analysis [25].

Moreover, the vertical location of the heterozygote for each SNP on the CEU panel shows a structural difference in the textile plot compared with the plots for the other populations. In an HWE population, the heterozygote on each axis is usually located in the middle of the homozygotes, except when there is a sampling bias (see Text S1 for details). Therefore, on the CEU panel, the heterozygote significantly deviating from the middle may suggest a systematic violation of HWE within the region. In addition, the extended homozygous stretch composed of the most frequent haplotype in the CEU population implies the existence of a long-range haplotype [26] in this region. In fact, the *LCT* gene is associated with a strong positive selection for an advantage to lactase persistence in the setting of dairy farming in the European population [4], this feature of the textile plot may provide a way to potentially leverage the plot for detecting the fingerprints of positive selections.

## Methods

The textile plot is applicable for any number of SNPs, but interpretation of the plot is hindered if too many SNPs are incorporated into one display. The appropriate locations of the genotypes on each vertical axis should be chosen so that all connected segments between adjacent genotypes are aligned as horizontally as possible, thus allowing for an understanding of the relationships between multiple-SNPs in a single display. The following subsections describe the genotype locations as well as the heuristic rearrangement of the axes in the textile plot.

### Encoding

The vector of the genotypes on SNP 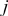 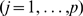 observed for individuals 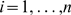 is denoted by 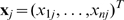, and the element 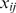 takes one of 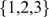. Here the homozygous genotypes are denoted by 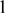 and 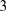, while the heterozygous genotype is represented by 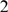. The coordinates (vertical positions) of genotypes 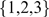 on SNP 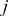 are 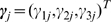. Therefore, the coordinates for 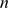 individuals on SNP 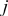 are represented by
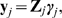
where the 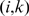-element of an 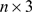 indicator matrix 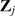 is
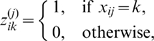
(1)for 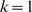, 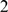 and 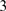. The vector 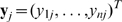 gives the coordinates of the 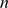 observations on the axis 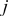. If an 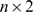 matrix 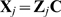 is defined by a 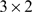 contrast matrix 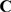 derived from a set of contrasts [27] such that 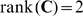 and the columns are all linearly independent of 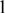, then 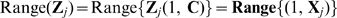. Therefore, the coordinate vector 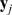 of the genotype vector 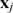 can be rewritten as
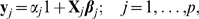
(2)where 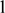 is a vector of ones. The location parameter 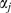 and the scale parameter vector 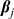 are chosen simultaneously.

As shown in [14], the textile plot can handle any qualitative and quantitative phenotype data in conjunction with SNP genotype data. For a qualitative phenotype, the encoding protocol is exactly the same as that of the SNP genotype in (1) for 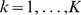, where 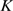 denotes the number of categories in any categorical variable (*e.g.*, 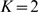 for binary traits). For a qualitative phenotype, such as height, weight, BMI (Body Mass Index), the matrix 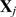 in (2) is simply replaced by a raw data vector instead of the encoded matrix. Note that the textile plot with QTL (quantitative trait loci) is not shown in this manuscript.

### Horizontalization criterion

**Figure 7:**
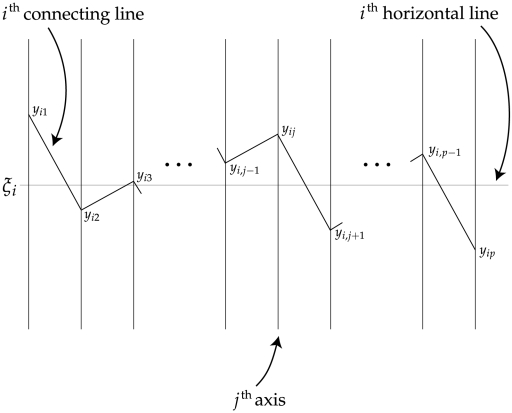
Degree of horizontalness in the textile plot. The polygonal line specified by 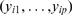 shows the observation for 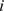
th individual, and horizontal line specified by 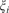 is the ideal coordinate for 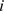
th individual. The squared sum of Euclidean distance between the polygonal line and the horizontal line on each axis is minimized for all individuals simultaneously.

The degree to which each connecting line on the textile plot is horizontal can be measured by the sum of the squared deviations from a horizontal line at level 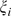 (referred to as the 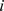
th horizontal line in Figure 7);
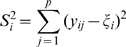
for the 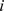
th line connecting the points at the levels 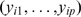. We selected 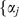, 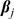; 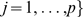 in order to minimize
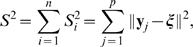
where 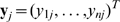 for 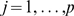. The vector 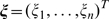 also must be chosen to minimize the sum of squares, because the levels 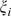, 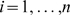 are unknown a priori.

In the textile plot, the sum of the squared deviations is not properly defined if there are missing values in the data. To reflect the existence of a missing value, we introduced weight vectors 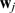, 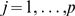 in which elements of zero or one are used to indicate missing values in 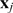, 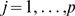. The 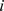
th element 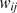 of 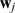 is 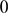 if the corresponding element 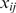 of 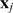 is missing; otherwise, 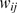 is 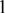. Using the notation 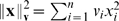 for the norm with a weighting vector 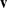, the sum of the squares is
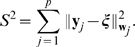
(3)


The horizontalization criterion and its minimization would be more appropriate for normally distributed data than for discrete or count data such as SNP genotype data. The position of each genotype on the axis exhibits the optimal position, and is determined based on the weighted 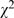 distance between any pair of SNP genotypes. It is identical with the *profile point* of each category corresponding to the first principal component axis in the correspondence analysis ([28], pp. 67). Similar to how the correspondence analysis is used for the dependence analysis of the multivariate categorical data, the horizontalization criterion of the textile plot can be applied to discrete data, such as SNP data, without loss of generality.

### The optimization problem and solution

In the textile plot, the sum of the squared deviations in (3) is minimized with respect to 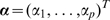, 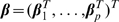 and 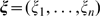. A constraint is needed to avoid trivial solutions such as 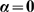, 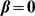 and 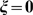. A natural constraint is to keep the total dispersion of the points on the textile plot constant; for example, the total dispersion can be set to equal the effective number of the points 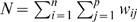, so that
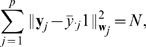
(4)where 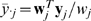 is the mean of the coordinate vector 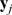 and 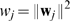.

A solution that minimizes (3) under constraint (4) is obtained by the Lagrange multiplier method (see Text S2 for details). Using the matrix notation
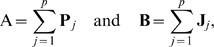
with projection matrices 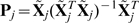 and 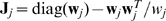 for 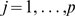, where 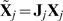 and 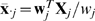, the following solution is obtained:
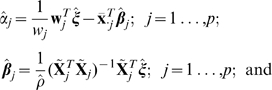
(5)The optimal solution in (5) is not uniquely determined when all the SNP pairs are independent of each other, implying that the textile plot cannot analyze the association between more than two SNPs that show pair-wise independence but the overall dependence. Therefore, the textile plot should be interpreted as a tool for “joint bivariate rather than multivariate” [28] analyses, similar to MCA.

### Rearrangement of SNP ordering

Whereas the heat map does an excellent job of displaying the pairwise LD for any pair of SNPs at any distance, the textile plot does not allow the LD between remote SNPs because of the structural defect in the parallel coordinate system. At the risk of losing the physical locations of SNPs within the LD block, we introduced several heuristic orderings of the vertical axes to overcome this issue in the textile plot.

A hierarchical clustering technique can be used to rearrange the SNP ordering. A natural choice for the distance between two adjacent SNPs is the mean absolute deviation 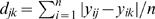 where the 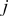
th and the 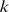
th SNPs are adjacent. Allowing for missing values, the distance becomes
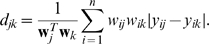
A clustering algorithm based on the above distance can be applied to order the SNPs. In fact, the ordered *single end-linkage* clustering algorithm [29] has been employed in the textile plot, providing both a heuristic ordering of the SNPs and a dendrogram. The distance 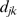 for any pair of SNPs 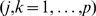 is required for the clustering algorithm.

### Software

Textile Plot software written in JAVA is available from http://www.stat.math.keio.ac.jp/TextilePlot/genetics/.

## Supporting Information

Figure S1 The vertical dispersion on axis *j*. Under Hardy-Weinberg equilibrium, the dispersion |γ*_j_*| is proportional to the *j*th element of eigenvector for the correlation matrix with respect to the largest eigenvalue.
(0.90 MB EPS)

Figure S2 LD display of the correlation coefficient (square root of *r* ^2^ statistics) for the same data set as in Figure 4. The large LD pattern is also confirmed in the middle of the plot. Note that the display was not constructed by using the HaploView software so that the shades of colors do NOT indicate the strength of *D′* statistic.
(25.53 MB EPS)

Figure S3 Textile plots of 10,000 subjects drawn at random from a HWE population according to the specific LD patterns. We assume two LD blocks, each of which has the same strength (*i.e.*, *D′* = 0.8 and *r* ^2^ = 0.64) and also the same strength between then (*i.e.*, *D′* = 0.1 and *r* ^2^ = 0.01). The MAF of each SNP is also fixed with a value of 0.4. We see that the vertical dispersion of the right hand side LD block (*e.g.*, |γ_6_|) increases, as the number of SNPs in the block increases.
(2.01 MB EPS)

Figure S4 Textile plots of 10,000 subjects drawn at random from a HWE population according to the specific LD patterns. We assume two LD blocks, where the relative scale of the strength of the LD within and between blocks are the same, but the absolute scale of the LD varies. The MAF of each SNP is fixed with a value of 0.4. We see that the proportion of the vertical dispersions (*e.g.*, |γ_1_|/|γ_10_|) for the two LD block does not change, while the absolute scale of the strength of LD is changed.
(2.13 MB EPS)

Figure S5 Textile plot of SNPs associated with chronic hepatitis B located in the entire *MHC* region. A large LD block can be confirmed in the region that extends over 28–34Mb on chromosome 6. Note that the plot shows only 203 SNPs in this region, because the set of SNPs has been narrowed down for the second stage of GWAS.
(4.00 MB EPS)

Figure S6 Pairwise *D′* (top) and *r* ^2^ (bottom) displays corresponding to Supplementary Figure 4. In *D′* display, a large LD block can be confirmed in the region that extends over 28–34Mb on chromosome 6, and it comprises a sub-block in strong LD located in a region surrounding *HLA-DPA1* and *HLA-DPB1* genes.
(38.21 MB EPS)

Figure S7 Textile plots of (a) all SNPs located in the region surrounding *HLA-DP* genes and SNPs and (b) with MAF>0.0. Note that, the SNP markers with MAF = 0.0 in the exon 2 of *HLA-DPA1* or *DPB1* are indispensable to determine HLA alleles.
(2.16 MB EPS)

Figure S8 Heat map displays for 14 SNPs surrounding *LCT* gene on chromosome 2 for the seven ethnic groups from HapMap data, corresponding to Figure 6. The left column indicates a series of LD displays based on *D′* statistics, while the right column indicates a series of LD displays based on *r* ^2^ statistics. The stronger LD in CEU population than the other population may imply the existence of long range haplotypes and thus it is a fingerprint of positive selections for an advantage to lactase persistence in the setting of dairy farming.
(2.78 MB EPS)

Table S1 LD statistics (*r* ^2^/*D′*) corresponding to Figure 1b.
(0.03 MB PDF)

Table S2 Comparison of haplotype frequencies.
(0.03 MB PDF)

Table S3 Mapping between SNPs in *HLA-DPA1* gene and *HLA-DPA1* alleles based on IMGT/HLA databases.
(0.03 MB PDF)

Table S4 Mapping between SNPs in *HLA-DPB1* gene and *HLA-DPB1* alleles based on IMGT/HLA databases.
(0.03 MB PDF)

Text S1 Mathematical interpretation of vertical dispersion and the location of the heterozygote.
(0.05 MB PDF)

Text S2 Solutions of optimization problem.
(0.05 MB PDF)
