# Supplementary material for: The Textile Plot: A New Linkage Disequilibrium Display of Multiple-Single Nucleotide Polymorphism Genotype Data
Source: PLoS One. 2010 Apr 27;5(4):e10207. doi: 10.1371/journal.pone.0010207 (PMC2860502; doi:10.1371/journal.pone.0010207)
Supplement: Table S1 — LD statistics (r 2/D′) corresponding to Figure 1b. (0.03 MB PDF) [file pone.0010207.s009.pdf]

Table 1. LD statistics (r^2/D') corresponding to Figure 1b.

		D statistic			
		0.24	0.15	0.08	0.01
	0.8/0.2	*	*	*	0.0039/0.2500
Allele Freq.	0.7/0.3	*	*	0.1451/0.8889	0.0023/0.1111
π_A/π_B	0.6/0.4	*	0.3906/0.9375	0.1111/0.5000	0.0017/0.0625
	0.5/0.5	0.9216/0.9600	0.3600/0.6000	0.1024/0.3200	0.0016/0.0400
