# Supplementary material for: The Textile Plot: A New Linkage Disequilibrium Display of Multiple-Single Nucleotide Polymorphism Genotype Data
Source: PLoS One. 2010 Apr 27;5(4):e10207. doi: 10.1371/journal.pone.0010207 (PMC2860502; doi:10.1371/journal.pone.0010207)
Supplement: Table S2 — Comparison of haplotype frequencies. (0.03 MB PDF) [file pone.0010207.s010.pdf]

Table 2. Comparison of haplotype frequencies.

Haplotype	Homo. Count	Freq. ($\sqrt{}$ Homo. Count)	Freq. (EM)
AAGA AAAG	171	.439	.437
GGAG AAAG	74	.289	.288
GGAG CGGA	66	.272	.269
AAAA AAAG	0	.0	.004
AAGA CGGA	0	.0	.002
Total		1.0	1.0

Observed three major haplotypes and two minor haplotypes are shown on the first column in conjunction with their counts of homozygous pairs on the second column. Relative frequencies of square root of the homozygous counts (the total sum is normalized to 1) and the corresponding haplotype frequencies estimated by using EM algorithm from the same data set are also shown on the third and forth columns. The square roots of the homozygous counts divided by the total sum are quite similar to the haplotype frequencies estimated by the EM algorithm, although the precise frequency of each minor haplotype cannot be obtained from the textile plot.
