# Supplementary material for: The Textile Plot: A New Linkage Disequilibrium Display of Multiple-Single Nucleotide Polymorphism Genotype Data
Source: PLoS One. 2010 Apr 27;5(4):e10207. doi: 10.1371/journal.pone.0010207 (PMC2860502; doi:10.1371/journal.pone.0010207)
Supplement: Table S3 — Mapping between SNPs in HLA-DPA1 gene and HLA-DPA1 alleles based on IMGT/HLA databases. (0.03 MB PDF) [file pone.0010207.s011.pdf]

Table 3. Mapping between SNPs in *HLA-DPA1* gene and *HLA-DPA1* alleles based on IMGT/HLA databases.

124	125	138	153	184	185	242	340	345	Allele
G	*C*	*G/C*	*G/A*	*A*	*T*	*A*	*A*	*C*	*0103
G	*C*	*G/C*	*G/A*	*C*	*A*	*G*	*G*	*T*	*0201
A	*T*	*C*	*G/A*	*C*	*A*	*G*	*G*	*T*	*0202
