# Supplementary material for: The Textile Plot: A New Linkage Disequilibrium Display of Multiple-Single Nucleotide Polymorphism Genotype Data
Source: PLoS One. 2010 Apr 27;5(4):e10207. doi: 10.1371/journal.pone.0010207 (PMC2860502; doi:10.1371/journal.pone.0010207)
Supplement: Table S4 — Mapping between SNPs in HLA-DPB1 gene and HLA-DPB1 alleles based on IMGT/HLA databases. (0.03 MB PDF) [file pone.0010207.s012.pdf]

Table 4. Mapping between SNPs in *HLA-DPB1* gene and *HLA-DPB1* alleles based on IMGT/HLA databases.

9	11	13	18	19	90	152	154	158	192	213	215	238	241	243	246	Allele
C	*T*	*T*	*G*	*G*	*C*	*G/C*	*C*	*G*	*A*	*A*	*G*	*A*	*A*	*G*	*G*	*0501
C	*T*	*T*	*G*	*G*	*T*	*T/G/C*	*A*	*G*	*G*	*A*	*G*	*G/T*	*G*	*C*	*A*	*0201
C	*T*	*T*	*G*	*G*	*C*	*G/C*	*C*	*G*	*G*	*A*	*G*	*G/T*	*G*	*C*	*A*	*0202
C	*T*	*T*	*G*	*G*	*T*	*T*	*C*	*G*	*A*	*A*	*G*	*G/T*	*G*	*C*	*A*	*0401
C	*T*	*T*	*G*	*G*	*T*	*T*	*A*	*G*	*A*	*A*	*G*	*G/T*	*G*	*C*	*A*	*0402
G	*G*	*A*	*T*	*T*	*T*	*T*	*A*	*C*	*G*	*G*	*A*	*A*	*A*	*G*	*G*	*0901
G	*G*	*A*	*T*	*T*	*T*	*T*	*A*	*C*	*A*	*G*	*A*	*A*	*A*	*G*	*G*	*0301
G	*G*	*A*	*T*	*T*	*T*	*T*	*C*	*G*	*G*	*A*	*A*	*A*	*A*	*G*	*G*	*1301
G	*G*	*A*	*T*	*T*	*T*	*T*	*A*	*C*	*A*	*G*	*A*	*A*	*A*	*G*	*G*	*1401
