# Supplementary material for: The Textile Plot: A New Linkage Disequilibrium Display of Multiple-Single Nucleotide Polymorphism Genotype Data
Source: PLoS One. 2010 Apr 27;5(4):e10207. doi: 10.1371/journal.pone.0010207 (PMC2860502; doi:10.1371/journal.pone.0010207)
Supplement: Text S1 — Mathematical interpretation of vertical dispersion and the location of the heterozygote. (0.05 MB PDF) [file pone.0010207.s013.pdf]

Text S1: Mathematical Interpretation of Vertical Dispersion and the Location of the Heterozygote

Here we employ the following set of contrasts for the genotype encoding such that

$$\begin{pmatrix} 1 \\ 2 \\ 3 \end{pmatrix} \to \begin{pmatrix} 0 & 0 \\ 1 & 0 \\ 1 & 1 \end{pmatrix}. \tag{1}$$

Since the solution $\hat{\beta}_j$, $j = 1, \ldots, p$ satisfies

$$(\tilde{\mathbf{X}}_j^T \tilde{\mathbf{X}}_j)^{-1} \tilde{\mathbf{X}}_j^T \sum_{k=1}^{p} \tilde{\mathbf{X}}_k \hat{\beta}_k = \hat{\rho} \hat{\beta}_j, \; j = 1, \ldots, p$$

(see Text S2 for details), we have

$$(\tilde{\mathbf{X}}_j^T \tilde{\mathbf{X}}_j)^{-1} \tilde{\mathbf{X}}_j^T \tilde{\mathbf{X}}_k \mathbf{1} = \sqrt{\frac{p_k(1-p_k)}{p_j(1-p_j)}} r_{jk} \mathbf{1}$$

and

$$(\tilde{\mathbf{X}}_j^T \tilde{\mathbf{X}}_j)^{-1} \tilde{\mathbf{X}}_j^T \tilde{\mathbf{X}}_k \begin{pmatrix} p_k - 1 \\ p_k \end{pmatrix} = \frac{p_k(1-p_k)}{p_j(1-p_j)} r_{jk}^2 \begin{pmatrix} p_j - 1 \\ p_j \end{pmatrix}$$

for $j = 1, \ldots, p$ under the assumption of Hardy-Weinberg equilibrium (HWE), where p_j is the allele frequency of SNP j, and r_{jk} is the correlation coefficient between SNP j and k, which is a square root of r^2 statistics. Let $\eta = (\eta_1, \ldots, \eta_p)^T$ and $\delta = (\delta_1, \ldots, \delta_p)^T$ respectively be an eigenvector of $\mathbf{R} = (r_{jk};\; j, k = 1, \ldots, p)$ and $\mathbf{R}^2 = (r_{jk}^2;\; j, k = 1, \ldots, p)$ associated with an eigenvalue θ and κ, we have

$$(\tilde{\mathbf{X}}_j^T \tilde{\mathbf{X}}_j)^{-1} \tilde{\mathbf{X}}_j^T \sum_{k=1}^{p} \tilde{\mathbf{X}}_k \mathbf{1} \frac{\eta_k}{\sqrt{p_k(1-p_k)}} = \mathbf{1} \frac{\sum_{k=1}^{p} r_{jk}\eta_k}{\sqrt{p_j(1-p_j)}} = \theta \mathbf{1} \frac{\eta_j}{\sqrt{p_j(1-p_j)}}$$

and

$$(\tilde{\mathbf{X}}_j^T \tilde{\mathbf{X}}_j)^{-1} \tilde{\mathbf{X}}_j^T \sum_{k=1}^{p} \tilde{\mathbf{X}}_k \begin{pmatrix} p_k - 1 \\ p_k \end{pmatrix} \frac{\delta_k}{p_k(1-p_k)} = \begin{pmatrix} p_j - 1 \\ p_j \end{pmatrix} \frac{\sum_{k=1}^{p} r_{jk}^2 \delta_k}{p_j(1-p_j)} = \kappa \begin{pmatrix} p_j - 1 \\ p_j \end{pmatrix} \frac{\delta_j}{p_j(1-p_j)}$$

for any p pairs of $\{\theta, \eta\}$ and $\{\kappa, \delta\}$. Therefore the solution of the minimization problem is attained at $\hat{\rho} = \max\{\theta, \kappa\}$ since there are totally $2p$ eigenvalues which cover all the local maximum of ρ. It is easily seen that $\max\{\theta\} \geq \max\{\kappa\}$ always holds true since $|r_{jk}| \geq |r_{jk}|^2$, the solution of β is equal to $\eta_j \mathbf{1}/\sqrt{p_j(1-p_j)}$ with respect to the contrast (1) except for a constant multiplication.

This leads to that the vertical dispersion γ_j (Supplementary Figure 1) is proportional to

$$\gamma_j \propto \frac{|\eta_j|}{\sqrt{2p_j(1-p_j)}} \approx \frac{\lambda}{p} \frac{\sum_{k=1}^{p} |r_{jk}|}{\sqrt{2p_j(1-p_j)}} \quad (j = 1, \ldots, p) \tag{2}$$

under HWE, where $\eta = (\eta_1, \ldots, \eta_p)^T$ is an eigenvector of the correlation matrix $\mathbf{R}$ associated with the largest eigenvalue λ. Therefore, the vertical dispersion of textile plot gives an optimal linear approximation of pairwise r^2 statistics by means of graphical representation. The approximation on the right hand side of the equation (2) indicates that the magnitude of γ_j is proportional to the sum of $|r_{jk}|$ for $k = 1, \ldots, p$ divided by squared root of expected heterozygosity $\sqrt{2p_j(1-p_j)}$. Note that, the approximation holds true when a part of SNPs are in sufficient LD, since R $\approx \lambda\eta\eta^T$. This implies that when observed SNPs are physically distributed sparsely and are nearly in LE, the interpretation of the vertical dispersion of the genotypes on each axis as stated above does not make sense.
