# Supplementary material for: The Textile Plot: A New Linkage Disequilibrium Display of Multiple-Single Nucleotide Polymorphism Genotype Data
Source: PLoS One. 2010 Apr 27;5(4):e10207. doi: 10.1371/journal.pone.0010207 (PMC2860502; doi:10.1371/journal.pone.0010207)
Supplement: Text S2 — Solutions of optimization problem. (0.05 MB PDF) [file pone.0010207.s014.pdf]

Text S2: Solutions of Optimization Problem

We shall provide the proof of obtaining the solution of the optimization problem. Let us define a Lagrangian function

$$L(\alpha, \beta, \xi; \lambda) = \sum_{j=1}^{p} \|\mathbf{y}_j - \xi\|^2_{\mathbf{w}_j} - \lambda\Big(\sum_{j=1}^{p} \|\mathbf{y}_j - \bar{y}_{\cdot j}\mathbf{1}\|^2_{\mathbf{w}_j} - N\Big)$$

with Lagrange multiplier $\lambda \geq 0$. Then, we have

$$\frac{1}{2}\frac{\partial L}{\partial \alpha_j} = \mathbf{w}_j^T(\mathbf{y}_j - \xi) - \lambda \mathbf{w}_j^T \mathbf{J}_j \mathbf{y}_j = \mathbf{w}_j^T \mathbf{y}_j - \mathbf{w}_j^T \xi,$$

since $\mathbf{J}_j \mathbf{w}_j = \mathbf{0}$. Therefore the solution $\hat{\alpha}_j$ satisfies

$$0 = \mathbf{w}_j^T \mathbf{y}_j - \mathbf{w}_j^T \xi = w_j \hat{\alpha}_j + w_j \bar{\mathbf{x}}_{\cdot j}^T \beta_j - \mathbf{w}_j^T \xi$$

which gives

$$\hat{\alpha}_j = \frac{1}{w_j}\mathbf{w}_j^T \xi - \bar{\mathbf{x}}_{\cdot j}^T \beta_j.$$

Then, we have

$$L(\hat{\alpha}, \beta, \xi; \lambda) = \sum_{j=1}^{p} \|\tilde{\mathbf{X}}_j \beta_j - \mathbf{J}_j \xi\|^2_{\mathbf{w}_j} - \lambda\Big(\sum_{j=1}^{p} \|\tilde{\mathbf{X}}_j \beta_j\|^2_{\mathbf{w}_j} - N\Big).$$

By differentiating with respect to β_j, we have

$$\frac{1}{2}\frac{\partial L}{\partial \beta_j} = \tilde{\mathbf{X}}_j^T(\tilde{\mathbf{X}}_j \beta_j - \mathbf{J}_j \xi) - \lambda \tilde{\mathbf{X}}_j^T \tilde{\mathbf{X}}_j \beta_j.$$

Therefore,

$$\mathbf{0} = (1 - \lambda)\tilde{\mathbf{X}}_j^T \tilde{\mathbf{X}}_j \beta_j - \tilde{\mathbf{X}}_j^T \mathbf{J}_j \xi$$

gives the solution of β_j such that

$$\hat{\beta}_j = \frac{1}{1 - \lambda}(\tilde{\mathbf{X}}_j^T \tilde{\mathbf{X}}_j)^{-1} \tilde{\mathbf{X}}_j^T \xi.$$

Then we have

$$L(\hat{\alpha}, \hat{\beta}, \xi; \lambda) = -\frac{1}{\rho}\xi^T \mathbf{A} \xi + \xi^T \mathbf{B} \xi + \lambda N.$$

The differentiation with respect to ξ gives

$$\frac{1}{2}\frac{\partial L}{\partial \xi} = -\frac{1}{\rho}\mathbf{A}\xi + \mathbf{B}\xi,$$

where $\rho = 1 - \lambda$. Therefore, the solution $\hat{\xi}$ is an eigenvector of $\mathbf{A}$ with respect to $\mathbf{B}$ with an eigenvalue $\hat{\rho}$. Then we choose $\hat{\xi}$ so that

$$\sum_{j=1}^{p} \|\mathbf{y}_j - \bar{y}_{\cdot j}\mathbf{1}\|^2_{\mathbf{w}_j} = \frac{1}{\hat{\rho}}\hat{\xi}^T \mathbf{B} \hat{\xi} = \frac{1}{\hat{\rho}^2}\hat{\xi}^T \mathbf{A} \hat{\xi} = N$$

is satisfied. The fact that

$$S^2(\hat{\alpha}, \hat{\beta}, \hat{\xi}) = \hat{\lambda} N = N - \hat{\rho} N$$

implies the largest eigenvalue $\hat{\rho}$ of $\mathbf{A}$ with respect to $\mathbf{B}$ attains the minimum of S^2, since $S^2 \geq 0$ is always satisfied.
